# Financial Distress and Homeownership: Evidence from SHARE Data covering 50- to 90-year-old Citizens of the Nordic and Baltic States from 2020 to 2022

**DOI:** 10.21203/rs.3.rs-10795121/v1

**Published:** 2026-08-25

**Authors:** Hans Gevers

**Affiliations:** Estonian Business School

**Keywords:** financial distress, personality traits, homeownership, SHARE

## Abstract

Homeownership is a societal and financial stronghold for people across the world. However, achieving or sustaining homeownership may come with a given cost. This study focuses on financial distress in relation to homeownership and other relevant factors like health. It relies on data from the Survey of Health, Ageing, and Retirement in Europe (SHARE) for citizens of the Nordic and Baltic States aged 50 to 90 years old. The analysis is completed with a logistic (N = 2,878) and ordered logistic (N = 7,030) estimator, which both model the change in the regressed variables from 2020 to 2022. The main findings reveal that homeownership exit alone does not relieve financial distress, yet results in an improvement in combination with a budget-for-food and income improvement. In contrast, a liabilities relief and a change in job situation appear to hinder a financial distress improvement. Overall, the findings can be regarded as relevant for policymakers’ quest for appropriate governmental action to handle citizens’ difficulties with making end meets which arise from sustaining homeownership.

## Introduction

Homeownership is one of the most common investments made by citizens across the world. It provides a given security and freedom, as well as an opportunity to accumulate wealth for retirement. Nevertheless, homeownership may also facilitate maintaining a debt burden at the expense of health, social life, and the like. This study explores the determinants of financial distress for homeowners aged 50 to 90 years old in the Nordic and Baltic States, and documents in particular the effects occurring when a homeownership exit takes place. Theoretically, the study is relatable to the Behavioural Life-Cycle hypothesis as defined by Shefrin and Thaler (1988). In brief, this model elaborates on how consumers are tempted to consume their income immediately and how their self-control and will-power facilitate securing financial means in retirement funds, homes, and the like. Furthermore, Shefrin et al.’s model regards household wealth as a combination of three mental accounts, i.e. the current spendable income, current assets, and future income. In this study, the first two are of interest, as constrained spendable income is expected to cause financial distress, while current assets may relieve this distress when liquidated. Shefrin et al. illustrate various predictions based on their hypothesis, and suggest that the impact of income and wealth mediated by homeownership is also testable. In this context, the following paragraphs explore the determinants of shortcomings in spendable income, i.e. financial distress, and their response to a release of ownership wealth.

### Literature Review

The existing literature provides several studies focusing on households’ financial distress. Jajko-Siwek (2024) identifies a wide range of features determining the financial well-being of SHARE respondents in Poland, Spain and Denmark. The latter is expected to be a country where retirees can more easily make ends meet, as well as where housing costs are more onerous. The Spanish and Polish appear to be more troubled by food expenditure. Across the three countries, household income is most relevant for financial well-being, followed by “past financial resources” like savings or real estate. Financial well-being also impacts overall well-being as documented by Netemeyer et al. (2018). They explore two dimensions of financial well-being, i.e. current money management stress and expected future financial security. Both are interrelated as well as exposed to behavioural influences like materialism, self-efficacy, self-control, risk aversion, and willingness to save. Efforts of employers and governments to support financial well-being may primarily concern the financial education of employees or citizens. On top of a time dimension, financial stress can be evaluated based on household type. In the Economics of Financial Stress, Sergeyev, Lian, and Gorodnichenko (2025) categorise a large sample of employees in the United States into naive and sophisticated households, with the former having the tendency to save less and subsequently being more vulnerable to financial stress. The sophisticated are expected to be more resilient to potential stress shocks impacting labour performance and the subsequent earnings. Sergeyev et al. primarily refer to policymakers to encourage saving to counter financial stress and its potentially subsequent poverty trap. Furthermore, financial distress is expected to align with findings in psychology regarding coping with stress. Hoeksema, Wisco, and Lyubomirsky (2008) note that various approaches can support coping with distress, e.g., denial, wishful thinking, escapism, problem-oriented, and the like. This coping capacity depends on individual neural, genetic, and cognitive conditions determining rumination on stressful situations. Additionally, financial distress is worsened at older ages by feelings of inability to self-manage as well as reduced participation in potentially costly activities. This statement is confirmed by Niedzwiedz, Pell, and Mitchell (2015) who additionally illustrate the overall strong relation between financial distress and well-being for varying welfare regimes in Europe. They conclude that policies ought to aim at increasing preparedness in early old age to cope with financial distress in order to reduce inequality at later life stages.

The existing literature also elaborates on the occurrence of financial distress in the context of homeownership. For homeowners, financial stress may arise from difficulties with paying back mortgages or loans. This implies a risk of distress arising from estimated future income as well as unforeseen costs for home maintenance or newborns. Furthermore, the income is dependent on the movements in the labour market and, subsequently, on the overall economic performance (Doling et al., 2007). The latter overarching interrelatedness suggests an important role for the government, which can ensure support for homeowners. Gobillon, Lambert, and Pellet (2022) document the subsidisation of home loans in France and find that for some, this implies moving to rural areas with an increased risk of deprivation, constrained job market access, and reduced social mobility. A similar housing question is raised by Burrows (2003) who finds that half of the poor in Britain are homeowners. He finds that poverty impacts the health of renters and owners differently. The former tend to be more socially excluded, while the latter more likely report mental health issues. The divide between both is relevant for assistance policies as sustaining homeownership in poverty may be less justifiable. However, the crucial question in this context remains how poverty would be defined. On top of safeguarding homeownership, various mechanisms may influence its transition. For example, Sharp, Whitehead, and Hall (2020) find that external family resources may overcome homeownership exit, while internal ones do not. Lastly, people with disabilities may in particular encounter difficulties with making ends meet, potentially in combination with homeownership, as they tend to be dependent on more sophisticated supportive programs. These programs rely on eligibility rules which may deny or provide incorrect access to benefits. Moreover, these programs are sensitive to time-dependent political choices. Based on this reasoning and backed by SHARE data of nine European countries, Morris (2021) finds that the odds for disability benefits recipients to experience deprivation double compared to non-recipients. As such, increased financial distress can be expected with the presence of limitations and worse health. To conclude, the existing literature provides several characteristics which are relevant for exploring the occurrence of financial distress in combination with homeownership.

### Data

The analysis in this study relies on data from SHARE (Bergmann et al., 2019; Börsch-Supan et al., 2013). The latter is a recurring health survey that inquires European citizens about the effects of health, social, economic and environmental policies. For the Nordic and Baltic States, i.e. Denmark, Sweden, Finland, Latvia, Lithuania, and Estonia, the data used is extracted from SHARE Waves 8 and 9 (SHARE-ERIC, 2024a, 2024b). From this dataset, only those who own a home in 2020 are taken forward to the subsequent survey year, i.e. 2022. Table 1 supports the selection of the observations.

As column three in Table 1 reveals, approximately 24 per cent of all responding homeowners in the Nordic and Baltic States have difficulty making ends meet in 2020. They represent approx. 18 per cent of the entire 2020 SHARE sample for these countries. For both Wave 8 and 9, the data is further processed to reflect the change from 2020 to 2022. After preprocessing, Wave 8 is reduced to 9,073 homeowners, as indicated in column five in Table 1, of which approx. 31 per cent have difficulties making ends meet in 2020. This dataset feeds the regressions with a maximum of 7,030 observations, given that missing values are accounted for. The following paragraph elaborates on the variables used as listed in Table 2.

**Figure 1:**
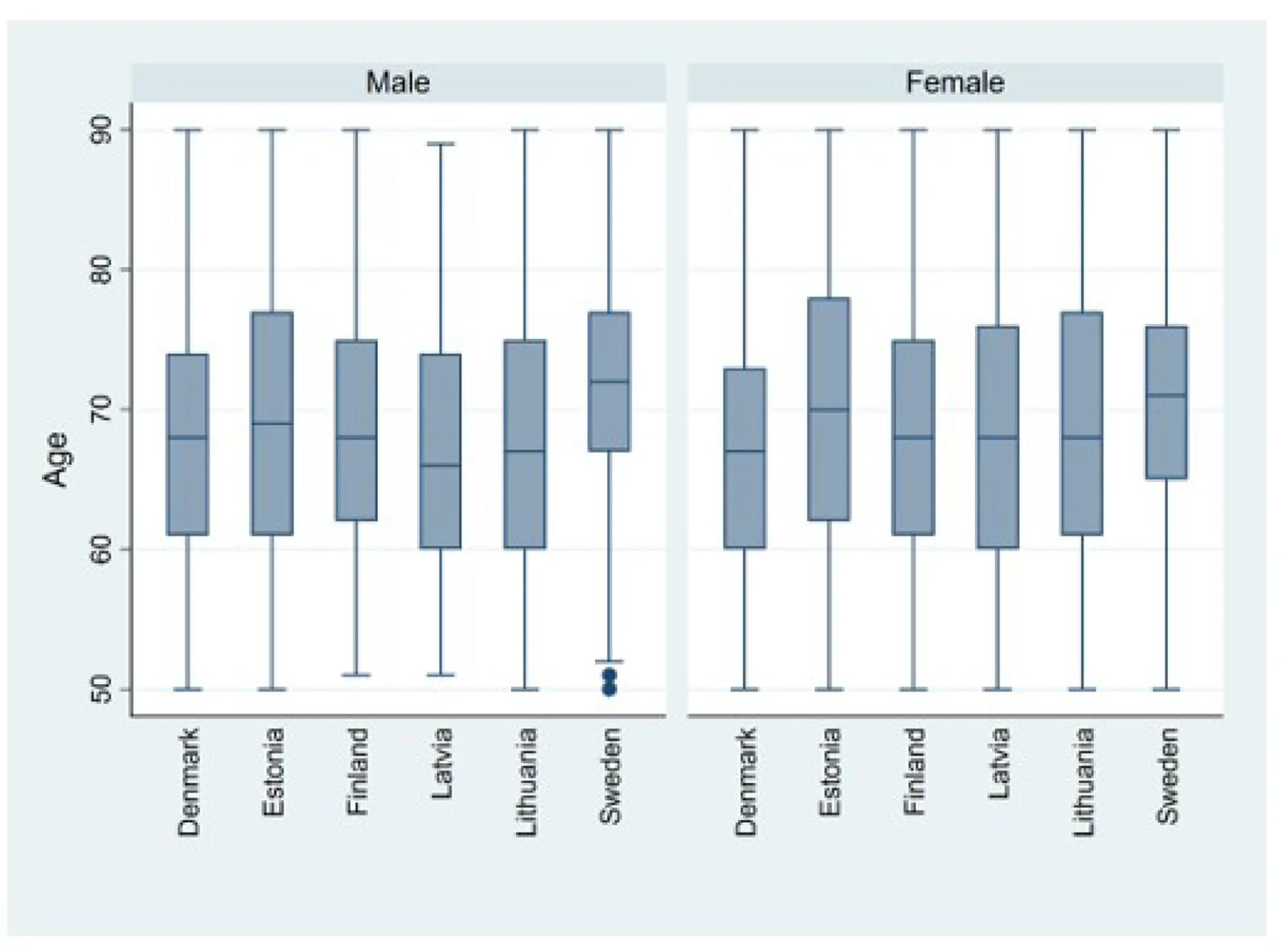
The age distribution of respondents in 2020 across countries per gender

The dependent variable is financial distress improvement, which is based on the respondent’s difficulty or easiness to make the ends meet. Its 2022 value is deducted from its 2020 value, which reflects the change in financial distress. For example, a switch from With great difficulty with the lowest value (1) to Easily with the highest value (4), results in a change of minus three. Overall, the scale for this change ranges from minus three to three. The scale is inverted and set as: strong negative change (0), average negative change (1), minimal negative change (2), no change (3), minimal positive change (4), average positive change (5), strong positive change (6). Similarly, twelve out of the sixteen dependent variables reflect a change across the period 2020 to 2022. For seven out of twelve dependent variables, the change is translated into a linear continuous variable, while for five out of twelve, the direction of the change is meaningless, implying that merely a change relative to no change, being a categorical, is informative. First, the continuous variables cover Depression relief, Limitations improvement, Health improvement, Income improvement, Liabilities relief, Food budget improvement, and Savings improvement. As for the calculation of the dependent variable, the difference between the observations of both periods for these variables results in a scale which reflects the most negative value being the worst possible outcome. Second, the categoricals Household, Housing, and Job situation are binary and merely indicate if the situation in 2022 changed relative to 2020. The variable Household reflects whether the respondents live single, with other singles, or as part of a couple. The variable Housing indicates if the respondent is an owner, member of a cooperative, (sub)tenant, or rent-free. It reveals the change in housing relative to 2020 for those who own a home in 2020. From the 2020 sample used, referred to in Table 1, a little more than five per cent are no longer homeowners in 2022. The variable Job situation reveals that in 2020 and 2022, more than half of the respondents in the used sample are retired, and more than a third is (self-)employed. Besides these dominant categories, the respondents may be unemployed, permanently sick, homemaker, or something else (e.g. student or volunteer). The last two categoricals are Paid work and Mortgage relief. Paid work indicates if the respondent changed status, being a negative change when stopped doing paid work, an unchanged situation when continued doing or not doing paid work, or a positive change when started doing paid work. The categorical Mortgage is calculated similarly. The final four out of sixteen independent variables are Age, Years of education, Gender and Country, which solely rely on the observations of 2022. On average a respondent is educated for almost thirteen years, with a minimum of zero and a maximum of 25. Moreover, the average respondent is 70 years old, with a minimum of 50 and a maximum of 90 years old. Figure 1 below illustrates the age distribution of the respondents across countries and by gender.

A last specification of the data variables is their correlation. Table 4 in Appendix tabulates the Spearman correlations between all regressed variables, i.e. those that primarily express the relative change from 2020 to 2022. The initial high correlations between the similar variables, Paid work and Job situation (above 0.600), no longer occur when only the relative change is taken into account. To conclude, the relationship between the dependent and independent variables is not exclusively one-way. For example, financial distress most likely impacts depression and health. To temper this shortcoming, the analysis solely focuses on respondents owning a house in 2020 who potentially changed housing in 2022. The change in the other dependent variables is expected to influence the financial distress level on top of or in combination with the change in housing.

## Methodology

The data is cross-sectionally modelled in Stata 19.5 Basic Edition and models the change in twelve variables as well as the 2022 observations of four ‘time-invariant’ variables. A change in the latter is assumed to be less informative than the most recent observed values. Two models are deployed, namely a logistic and an ordered logistic estimator. The logistic estimator regards financial distress as a binary variable with a negative change as zero and a positive change as one. The ordered logistic estimator takes into account the ordered levels of financial distress and preferably respects the proportional odds assumption. For both estimators, robust standard errors are reported.

## Results

The estimation results are revealed in Table 3 on the following page.

Both estimators converged, solely the Ordered logistic estimator appears to violate the proportional odds assumption. Both the Likelihood-ratio test and Score test support a violation, while the Wald test suggests otherwise. This implies that the results are potentially inaccurate or unreliable. As such, the logistic estimator is used as a benchmark.

The results in Table 3 identify the relevant influences on financial distress, i.e. the ability to make ends meet. As such, Depression relief increases the odds of distress improvement, as confirmed by both the Logit and Ologit estimator (respectively Odds ratio (OR) 1.009, p<.05 and OR 1.006, p<.05). The main effect of Liabilities relief reveals higher yet not significant odds. The interaction effect of Liabilities relief with Changed housing surprisingly notes lower odds to report financial distress improvement (Logit OR 0.900, p<.05). The fact that when a home is liquidated may not suffice to relieve liabilities, may explain this finding. Additionally, Limitations improvement increases the odds of relieving distress (Logit OR 1.017, p<.05 and Ologit OR 1.011, p<.05). It has no significant impact in interaction with Changed Housing. Similarly, Health improvement triggers higher odds to report distress improvement (Logit OR 1.008, p<.10 and Ologit OR 1.005, p<.10). Budget for food improvement solely reports insignificant odds, yet in interaction with Changed Housing it increases the odds to report financial distress improvement (Logit OR 1.110, p<.05 and Ologit OR 1.067, p<.05). Also Income improvement relieves financial distress (Logit OR 1.044, p<.05 and Ologit OR 1.014, p<.05). Additionally, when Housing is changed it may have an even stronger effect (Logit OR 1.286, p<.10). Improvement in savings reports no significant impact. However, the change in Mortgage significantly impacts financial distress, primarily when it regards a negative change (Logit OR 0.479, p<.01 and Ologit OR 0.713, p<.05). A positive change suggests a less explicit decreased odds to report a distress improvement (Logit OR 0.686, p<.10). A possible explanation is that the burden of the mortgage persists regardless of relief. A change in Household composition tabulates decreased odds, yet these are not significant. A change in Job situation itself as well as in interaction with Changed Housing triggers lower odds to rate distress improvement (respectively: Logit OR 0.812, p<.10; Ologit OR 0.426, p<.05). Given that the majority of respondents are retirees and the larger minority (self-)employed, the decreased odds may arise from the latter resulting from a homeownership exit combined with permanent sickness. This explanation neglects employment loss, as this is more likely to be captured by the change in Paid work. A negative change, i.e. no longer doing paid work, constrains financial distress improvement (Logit OR 0.687, p<.01 and Ologit OR 0.795, p<.01), while a positive change triggers distress improvement (Logit OR 1.813, p<.05 and Ologit OR 1.320, p<.10). Besides the influence of the variables reflecting the change of 2020 to 2022, four variables are included to explain the financial distress change by personal characteristics. As such, ageing increases the odds to report distress improvement (Logit OR 1.011, p<.05 and Ologit OR 1.007, p<.05). This finding may relate to respondents’ expectations regarding the sufficiency of accumulated wealth to cover future life demands. Furthermore, Years of education and Gender tabulate non-significant and non-congruent odds across estimators. A meaningful impact of both is thus less likely. Lastly, the odds to report financial distress improvement are, relatively to Denmark, higher for Estonia, Finland, and Lithuania (respectively: Logit OR 1.300, p<.10 and Ologit OR 1.277, p<.01; Logit OR 1.819, p<.01 and Ologit OR 1.407, p<.01; Logit OR 1.704, p<.01 and Ologit OR 1.418, p<.01). These differences appear not to be driven by Changed Housing (not tabulated), yet may arise from imbalances in the data. However, an imbalance ought to imply that the findings align with the distribution of observations across countries. The distribution is as follows: Denmark covers approx. 18 per cent, Estonia 31 per cent, Finland 9 per cent, Latvia 11 per cent, Lithuania 15 per cent, and Sweden 16 per cent of the observations. An important final note for interpreting the results is that the main effects of Liabilities relief and Budget for food improvement are not significant, while their interaction effects are. Furthermore, the main effect of Changed Housing is likely relevant, yet amounts to zero (Logit OR 0.000, p<.10). This implies that homeownership exit itself rather relates to worse financial distress, yet that a less negative outcome may be obtained when other influences simultaneously improve.

**Figure 2:**
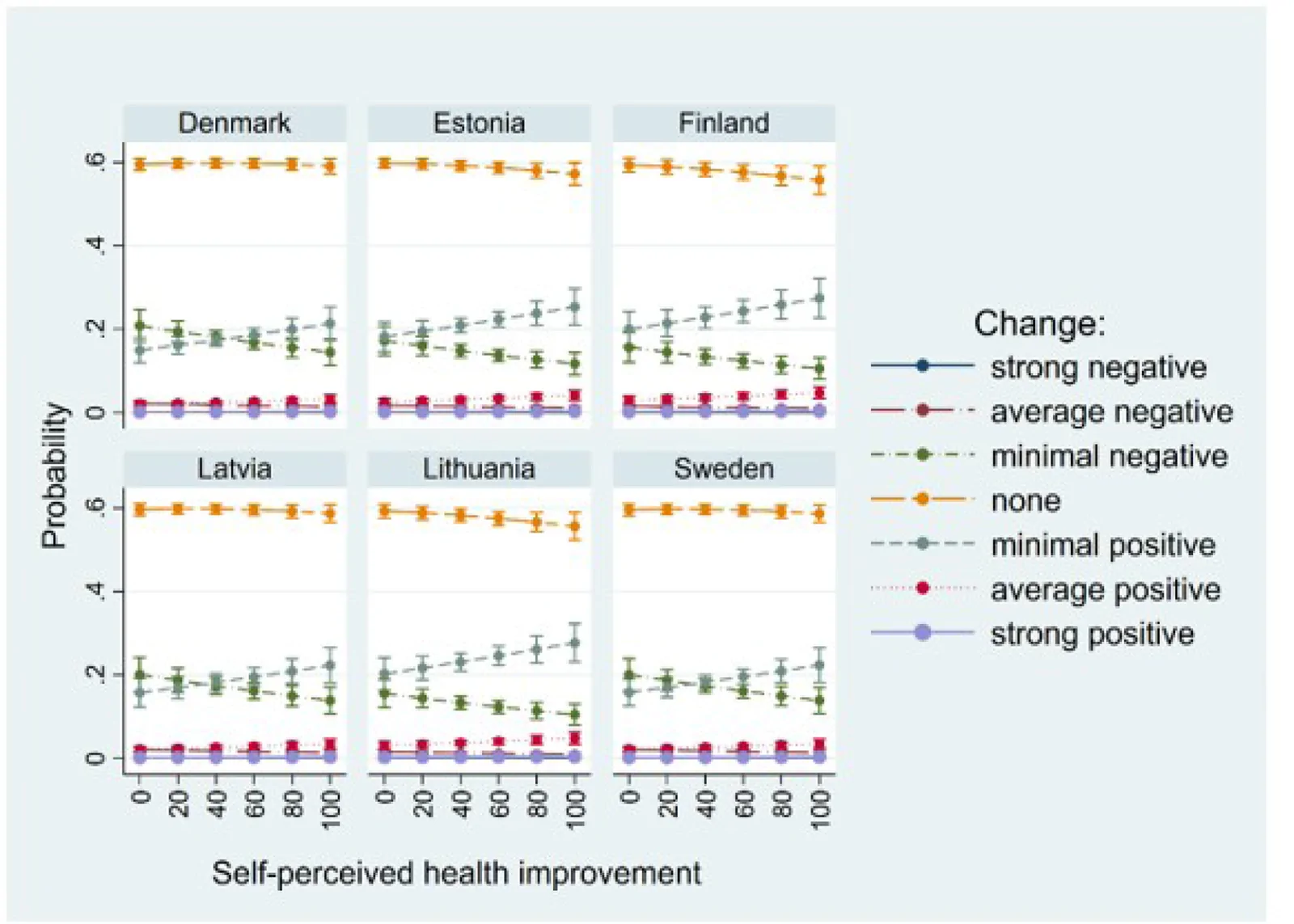
Margins Self-perceived health improvement and Financial Distress per Country Note: 95% Confidence Intervals are plotted. Improvement is scaled across countries.

On top of the regression output, Figure 2 on the following page plots, illustratively, per country the margins of financial distress given a change in self-perceived health improvement. This figure relies on the predictions of the Ologit estimator, which is expected to be less reliable due to the violation of the proportional-odds assumption. As such, the figure merely suggests how effects may occur at a different tipping point per country.

Figure 2 illustrates how, for Denmark, Latvia, and Sweden, the probability of scoring a minimal positive change in financial distress exceeds the probability of scoring a minimal negative one when self-perceived health improves by 40 per cent. For Estonia, Finland, and Lithuania, the probability of scoring a minimal positive change in financial distress is consistently higher than a minimal negative one. Regardless of the plausibility and accuracy of this finding, it suggests an a priori difference between countries in the health improvement needed to tip over the change in financial distress from negative to positive. To conclude, the findings support that homeownership exit in combination with other financial aspects relieves financial distress. Improvements in health and depression similarly relieve distress, yet their effect is not dependent on homeownership exit. Financial distress improvement is primarily constrained by a higher mortgage burden and the loss of paid work.

## Discussion

Despite its informativeness, the study comes with some limitations. First, the estimation procedure focuses on homeowners in 2020 who potentially exit their home in 2022, and, subsequently, on revealing the specific influences determining the change in the homeowners’ self-perceived level of financial distress. However, several independent variables, like health or income, do not explicitly determine financial distress, and may even be explained by it. Second, the Logit estimator neglects the category No change in the dependent variable, which limits the number of observations as well as impacts the comparability of both estimators used. Further research may focus on further unravelling the complexity of the gains of homeownership exit in times of financial difficulty.

## Conclusion

This study reveals that financial distress improvement or relief is supported by homeownership exit when combined with improvement in available income as well as budget for food. In contrast, homeownership exit in combination with liabilities relief or a change in job situation decreases the odds to score a financial distress improvement. These findings suggest that homeownership exit may result in more available means, yet also may not be sufficient to cover the liabilities or may result in a lower paycheck. Regardless of homeownership exit, financial distress improvement appears directly and positively related to activity limitation reduction, as well as physical and mental health improvements. Increases in mortgages and loss of paid work appear to most explicitly hinder the improvement of financial distress. Overall, the study provides insight into the mechanisms that steer financial distress on the occurrence of a homeownership exit by 50- to 90-year-olds in the Nordic and Baltic States. In particular for policymaking, the assumption that homeownership exit solves poverty, i.e. sustained difficulties to make ends meet, is a crucial question for allocating government resources as well as for outlining government interventions.

## Supplementary Material

Supplementary Files

This is a list of supplementary files associated with this preprint. Click to download.

• Appendix.docx

## Figures and Tables

**Table 1:** Financial Distress among homeowners in the Nordic and Baltic States

	Wave 8 SHARE 2020		Used sample 2020	
	absolute	relative	absolute	relative
With great difficulty	3,140	5.18	610	6.72
With some difficulty	11,376	18.76	2,220	24.47
Fairly easily	14,863	24.50	2,903	32.00
Easily	17,026	28.07	3,340	36.81
Total	46,405	76.51	9,073	100.00

**Table 2:** Descriptives

Variable	Unique	Mean	Minimum	Maximum
Financial distress improvement	7	3.091	0	6
Depression relief	20	51.746	0	100
Limitations improvement	18	49.379	0	100
Health improvement	9	49.013	0	100
Income improvement	4,336	65.590	0	100
Liabilities relief	636	47.231	0	100
Food budget improvement	1,177	37.237	0	100
Savings improvement	3,440	18.596	0	100
Household	2	0.285	0	1
Housing	2	0.270	0	1
Job situation	2	0.328	0	1
Paid work	2	-	0	1
Mortgage	3	-	0	2
Years of education	29	12.562	0	25
Age	41	70.400	50	90
Gender	2	-	1	2
Country	6	-	1	6

Number of observations: 7,030

**Table 3:** Odds ratios explaining the Financial Distress improvement among homeowners in the Nordic and Baltic States

VARIABLES	Logit	Ologit
Depression relief	1.009**	1.006**
	(0.004)	(0.003)
Liabilities relief	1.002	1.003
	(0.010)	(0.007)
Limitations improvement	1.017**	1.011**
	(0.007)	(0.004)
Health improvement	1.008*	1.005*
	(0.004)	(0.002)
Budget for food improvement	1.011	1.007
	(0.008)	(0.004)
Income improvement	1.044**	1.014**
	(0.022)	(0.007)
Savings improvement	0.993	0.998
	(0.018)	(0.008)
Mortgage: No change (base)		
Negative change	0.479***	0.713**
	(0.126)	(0.118)
Positive change	0.686*	0.844
	(0.146)	(0.094)
Household: No change (base)		
Changed	0.909	0.979
	(0.133)	(0.100)
Job situation: No change (base)		
Changed	0.812*	0.883
	(0.098)	(0.069)
Paid work: No change (base)		
Negative change	0.687***	0.795***
	(0.099)	(0.070)
Positive change	1.813**	1.320*
	(0.496)	(0.205)
Housing: No change (base)		
Changed	0.000*	0.257
	(0.000)	(1.434)
Changed Housing # Liabilities relief	0.900**	0.969
	(0.048)	(0.027)
Changed Housing # Budget for food improvement	1.110**	1.067**
	(0.050)	(0.030)
Changed Housing # Income improvement	1.286*	1.040
	(0.196)	(0.064)
Changed Housing # Changed job situation	0.484	0.426**
	(0.266)	(0.182)

Age	1.011**	1.007**
	(0.005)	(0.003)
Years of education	1.001	0.993
	(0.011)	(0.007)
Gender: Male (base)		
Female	0.995	1.007
	(0.082)	(0.049)
Country: Denmark (base)		
Estonia	1.300*	1.277***
	(0.174)	(0.089)
Finland	1.819***	1.407***
	(0.326)	(0.125)
Latvia	1.060	1.033
	(0.183)	(0.088)
Lithuania	1.704***	1.418***
	(0.266)	(0.111)
Sweden	1.029	1.028
	(0.165)	(0.073)

Number of observations	2,878	7,030

Notes:

Logit = logistic estimator with dependent negative change (0) or positive change (1) while excluding no change. Ologit = ordered logistic estimator with dependent strong negative change (0), average negative change (1), minimal negative change (2), no change (3), minimal positive change (4), average positive change (5), and strong positive change (6). Age, Years of education, and Gender represent the values of 2022, which are potentially changed, while the values for Country are the same in both years. Only the significant interaction effects of Housing with the other variables indicating the change over time are tabulated.

Robust standard errors in parentheses.

*** p<0.01,

** p<0.05,

* p<0.10

## Data Availability

To provide insight into the longitudinal analysis completed in this article, do- and log-files of Stata are available via https://github.com/hansgevers/financialdistress.
